# Auricular Acupressure Can Modulate Pain Threshold

**DOI:** 10.1155/2015/457390

**Published:** 2015-07-07

**Authors:** Antonietta Santoro, Stefania Lucia Nori, Letizia Lorusso, Carmine Secondulfo, Marcellino Monda, Andrea Viggiano

**Affiliations:** ^1^Department of Medicine and Surgery, University of Salerno, 84084 Fisciano, Salerno, Italy; ^2^Department of Experimental Medicine, Second University of Naples, 80138 Naples, Italy

## Abstract

The objective of our study was to investigate if auriculotherapy (AT) can modulate pain threshold. In our experiments, AT consisted of placing *Vaccaria* seeds over the “fingers point” of one ear. Two groups of healthy volunteers were enrolled for the study. Each subject was asked to perform an autoalgometric test developed by our group on three occasions: before, 1 hour after, AT and 24 hours after AT. Participants of the first group received a 2-minute long session of AT, while participants of the second group received a 2-minute long session of sham treatment, consisting of a puncture/massage above the skin of the neck. The autoalgometric test consisted of applying an increasing pressure with the finger-tips and finger-backs of four fingers by the subjects themselves (i.e., eight sites were evaluated) against a round-shaped needle for two times: until a minimum pain sensation (first time, minimal test) or a maximally tolerable pain sensation (second time, maximal test). Our results showed a significant higher pain threshold in the maximal test at 24 hours after AT compared to sham treatment. This result indicates for the first time that AT can increase pain tolerability, rather than affecting the minimal pain threshold.

## 1. Introduction

*Auriculotherapy: A Brief Overview*. Auriculotherapy (AT) is a treatment method aimed at normalizing body's dysfunction through the stimulation of definite points on the surface of the ear. AT is a treatment diffused all over the world, and its patterns follow the principles of Chinese acupuncture. In Chinese traditional medicine it was believed that the stimulation of auricular acupoints could regulate self-energy balance alleviating pathological conditions and pain through restoring flow energy into the body [1, 2]. In the last decades, an increasing number of data provided scientific data corroborating the efficacy of AT. A significant contribution was given by Nogier [3], who published the somatotopic map pattern of the external ear on the bases of studies carried out by the French acupuncturist Georges Soulie de Morant [4]. Nogier argued that the ear surface is the image of an inverted fetus in the womb and proposed that points in the body would correspond precisely with fetal representation in the auricle [5]. Nogier's map was comparable to the neurological homunculus for the human cerebral cortex by Penfield and Rasmussen [6] thus suggesting that AT could be based on the principles of reflexology rather than an energetic-based stimulation. Later in 1980, Oleson et al. published an important paper that became a milestone in ear acupuncture [7]. To assess the claim by French and Chinese ear acupuncture that there was a somatotopic organization of the body represented upon the human auricle, Oleson and colleagues examined 40 subjects. Patients were medically examined to determine areas of their body where there was musculoskeletal pain. Each patient was then draped with a sheet to hide any visible physical problems. The physician conducting the auricular diagnosis had no prior knowledge of the patient's medical condition but simply examined the patient's ear for areas of elevated skin conductivity or tenderness according to the French's and the Chinese's method of diagnosis. The concordance between the established medical diagnosis and the auricular diagnoses was 75.2%. These results supported the hypothesis that there was a somatotopic map of the body represented on the ear surface, but it represented definite areas not meridian lines or other energetic concepts. However, the use of AT to treat diseases raised several skepticisms, as it was unclear how AT could affect distal organs. It is now generally accepted that the stimulation of a specific region of external ear does not flow directly to distal organs, but the stimulation of a peripheral reflex on the ear surface travels along neuron fibers from the auricle to the brain, from the brain through the spinal cord, and from spinal nerves to the correspondent region of the body [8]. This complex pathway of communication along the nervous system could be responsible for reducing pain in distal organs. Therefore, AT can be considered as a clinical procedure for stimulating peripheral reflexes, which in turn activates the central brain pathways, thus inhibiting the maladaptive reflexes that contribute to pain and pathological disorders [8, 9].

*Auriculotherapy in the Treatment of Pain*. From the ancient traditional medicine to our days, AT has been considered an alternative therapy to treat pain. Types of AT include auricular acupuncture, electroacupuncture stimulation, and acupressure. The former two approaches include needle insertion or application of electrical stimulation to ear acupoints, while auricular acupressure utilizes very little plant seeds taped on the patient's ear lobe for acupoint stimulation. Studies using AT have strongly suggested promising effects in the pain management of several diseases, including low-back pain [10–12], hip fracture [13], dysmenorrhea [14, 15], polycystic ovary syndrome [16], and postoperative pain [17, 18]. A recent meta-analysis of randomized controlled trials, comprising studies carried out up to December 2008, suggested that AT can be effective for the treatment of various types of pain. In particular, AT reduced analgesic use in perioperative pain and decreased pain intensity in acute and chronic pain compared with control groups [19]. By evaluating larger sample size and more randomized controlled trials (up to 2013), Yeh and collaborators [20] expanded and corroborated data of the previous study and, in terms of the efficacy of the different treatment methods, showed that auricular acupressure exhibited the largest strength of evidence for pain relief, followed by auricular acupuncture. Electroacupuncture stimulation did not show significant evidence for efficacy, but few subjects were included in the analysis.

The mechanisms by which AT exerts its therapeutic effects are still unclear, but it has been demonstrated that electrical stimulation of rabbit auricular lobe in the region corresponding to the jaw and teeth in humans produced a significant decrease in behavioral reflexes and in cortical potentials evoked by electrical stimulation of the tooth pulp [21]. The opiate antagonist naloxone blocks both stimulation-produced analgesia from brain stimulation and analgesia from stimulation of acupuncture points, thus suggesting that a descending pain system able to inhibit pain perception can be associated with the endorphinergic pathway in the brain and spinal cord [22, 23]. This hypothesis has been corroborated by the findings of higher levels of endorphins after auricular stimulation [24–26]. Subsequent research has also suggested that cortisol, serotonin, and norepinephrine also play a significant role in these neural pathways which regulate pain [27]. Interestingly, recent data show that auricular point acupressure during the treatment of low-back pain decreased serum level of some cytokines such as IL-4 and IL-10 while TNF-*α*, IL-2, and IL-6 were increased [28]. This observation suggests that AT can also modulate the inflammatory process in chronic low-back pain treatment. On the other hand, Chan et al. [29] found an increased concentration of the spinal antinociceptive neurotransmitter substance P in skin tissue samples derived from acupuncture points in anesthetized dogs, thus suggesting that there is a difference in the neurochemical profile between acupuncture points and control points. Concerning the molecular mechanisms involved in the nociceptive signaling pathways modulated by AT, it has been reported that AT by acupuncture-based techniques exerts its therapeutic effects causing a decrease in activity of the p38 signaling pathway in the spinal cord [30], an increase in NF-*κ*B activity [31, 32], and a negative regulation of nerve growth factor (NGF) [32–34].

Although increasing data indicate promising benefits of AT in pain management of several pathologies, it is still unclear whether it can affect pain threshold. Therefore, we stimulated the ear surface by using* Vaccaria* seeds and measured pain threshold after such AT treatment by using an autoalgometer [35]. Briefly, the autoalgometer is a pressure detector put in a small box and connected to a personal computer equipped with custom software able to take force readings (10 samples/sec). On the top of the box there is a metal needle fixed to a force transducer. People can apply a gradually increasing pressure on the needle with their finger until they feel a minimal or a maximal pain sensation and the apparatus is able to record these data, determining pain threshold [35]. To date, pain threshold can be evaluated also with other methods including cuff algometry [36], skin or oral pressure algometry [37–39], and algometry with electric stimulation [36, 37]. However, all these methods require to be performed by expert investigators and the stimulus intensity requires to be gradually increased by a tester or by an electronic device while the patient reports his/her sensation. Our method does not require a particular expertise and the subject under examination applies himself/herself the pressure on the blunt tip that evokes pain. This has the advantage that only the subject under evaluation can control the pressure applied to the tip, so that the experimenter could not physically interfere with the procedure.

## 2. Materials and Methods

### 2.1. Method

The design of the study consisted of comparing the pain threshold of a group of healthy peoples receiving an auricular acupressure treatment by using* Vaccaria* seeds with that of another group of healthy people (matched for sex, age, weight, and height with the previous group) receiving a “sham treatment” (acupressure on the neck). For each group, the pain threshold was evaluated with an autoalgometer (described below) on three different occasions: (1) before the treatment, (2) 1 hour after the treatment, and (3) 24 hours after the treatment. On each occasion, both a “minimal pain” threshold and a “maximal pain” threshold were evaluated as described below.

### 2.2. Participants and Setting

16 healthy volunteers were recruited among young people attending the University of Salerno (Fisciano, Italy), and they were divided into two groups, with ages between 20 and 24 years (8 women and 8 men), weights between 50 and 93 kg, and heights between 152 and 184 cm (Table 1). All participants did not have any piercing on the point of interest for auricular acupressure.

### 2.3. Measures

**Figure 1:**
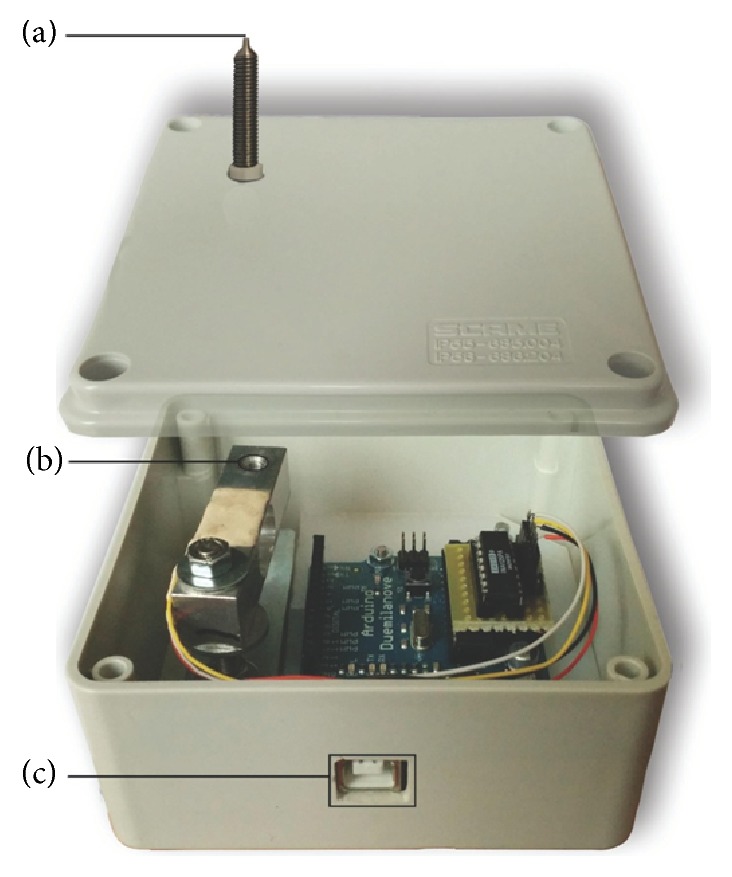
The autoalgometer. The device consisted of a round metal tip of 1 mm in diameter (a) fixed to a load-cell (b). The digital reading of the force applied to the metal tip was transmitted to a PC through a USB link (c).

Pain threshold was evaluated by using an autoalgometer. The autoalgometer is a pressure detector (pressure gage device) put in a small box of this size: 5.7 cm × 10.4 cm × 10.6 cm (Figure 1). In the back side of the box there is a USB port that links the device to a PC. On the top of the box there is a metal needle of 1.0 mm in diameter fixed to a force transducer (cell load). After the driver has been installed, custom software takes force readings (10 samples/sec) and saves them for subsequent analysis. Before the experiment was conducted, to evaluate the pain threshold, people were instructed to apply a gradually increasing pressure on the metal tip with their finger-tips or finger-backs until they felt a sensation that they would describe as the “minimal pain intensity that is possible to feel”; then they took off the finger from the metal tip. The software recorded the pressure applied to the metal tip all over the time of the test with a sampling frequency of 10 samples/sec; thus, the maximal value reached during the test was defined as the pain threshold. For each subject, the mean value from eight autoalgometric tests performed on eight different points was evaluated and considered for subsequent analysis; these points were the tip and the back of the second, third, fourth, and fifth fingers. After completing these 8 “minimal tests,” the participants performed 8 more “maximal” tests, repeating the same procedure, as before, but increasing, this time, the pressure over the metal tip until they felt a sensation that they would describe as the “maximal intensity of pain they would tolerate.” Thus, also the mean value of these 8 maximal tests was considered for subsequent analyses. In summary, each autoalgometric examination yielded two variables: a minimal pain threshold (the mean value from 8 minimal tests) and a maximal pain threshold (the mean value from 8 maximal tests).

### 2.4. Study Procedure

**Figure 2:**
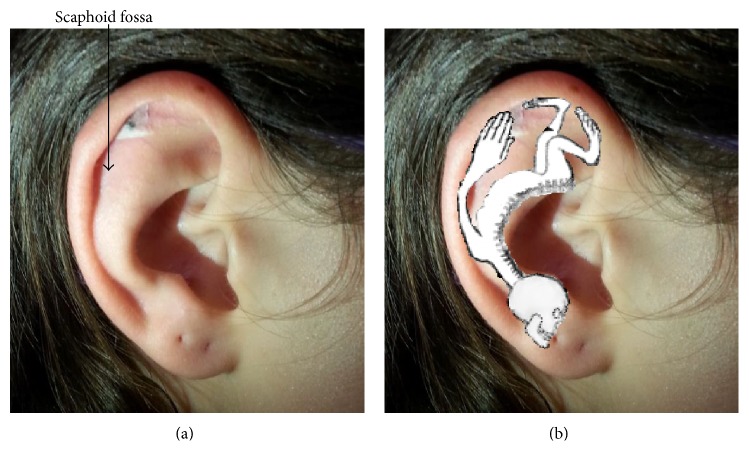
Location of* Vaccaria* seed on the surface of subject's earlobe (a). Somatotopic map of the ear showing the correspondence of fingers and fingertips with the stimulated acupoint (b).

The first day, from 14:00 to 19:00, each one of the participants underwent an autoalgometric examination, before any treatment. After this first examination, a seed of* Vaccaria* was applied on the top of the scaphoid fossa of one earlobe in the region corresponding to the finger-tips and finger-backs in Nogier's somatotopic map (Figure 2; treated group) or on the neck (control group).* Vaccaria* seed was applied by Dr. Nori, a qualified and trained physician [16, 32]. The acupoint was selected by searching the point most sensitive to a probe (tip tweezers) and tender to palpation. A gentle pressure with the thumb was applied to this seed, making a circular anticlockwise movement for two minutes with no further manipulation by the subject [40]. A second autoalgometric examination was then done after one hour, and a third examination was done after 24 hours by the application of the seed.

### 2.5. Statistical Analysis

Data are presented as means ± S.E. For each subject the values at 1 hour and 24 hours were normalized (divided) by the values obtained before the treatment; these normalized values were considered for the statistical analysis. Statistical significant differences between groups were evaluated with the analysis of the variance (ANOVA); multiple pairwise comparisons were done with Student's *t*-test with Bonferroni correction for the *P* value.

## 3. Results

**Figure 3:**
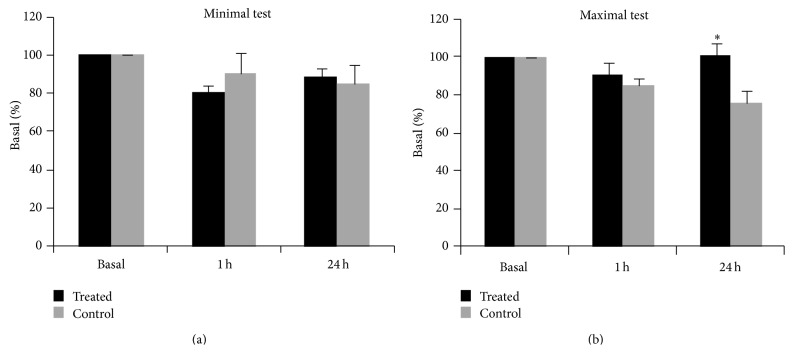
Mean ± S.E. values for the autoalgometric tests obtained before (basal) and 1 hour and 24 hours after the auricular acupressure (treated) or the placebo treatment (control). The values are expressed in percentage compared to the basal values. The asterisk indicates a significant difference compared with the control group at the same time point (*P* < 0.05).

The mean pain threshold values obtained with the autoalgometer for both treated and control groups are reported in Table 2. One hour after the treatment with* Vaccaria* seed there was no significant difference in the pain threshold between the two experimental groups, either in the minimal test or in the maximal test. 24 hours after the treatment, there was an increase in pain threshold in the treated group compared to the control group for the maximal test, but not for the minimal test (Figure 3). A two-way ANOVA, considering the variables treatment (with two levels: treated and controls) and time (with three levels: time 0, 1 hour, and 24 hours), demonstrated a significant effect for the treatment × time interaction (the *F*-value with 1 and 27 degrees of freedom was 11.7; the *P* value was <0.01); the post hoc test demonstrated a significant difference between the treated and the control groups at 24 hours from the treatment (*P* < 0.05).

## 4. Discussion

Our study indicates that AT is able to increase pain threshold in healthy volunteers by using autoalgometry, a novel method to test pain sensation [35].

There are many techniques to evaluate pain threshold but all of them require the stimulus intensity to be gradually increased by a tester or by an electronic device while the subject reports his/her sensation [39, 41, 42]. On the contrary, our method avoids any external interference; in fact, the autoalgometry procedure permits that only the subject under evaluation can control the pressure applied to the tip, so that the experimenter could not physically interfere with the procedure. Anyway, a major limit of this procedure is that the pain threshold can be evaluated only on the fingers, because it is impractical to ask people to finely control the pressure applied against a tip with other parts of the body. Our results also showed that effect of AT was significant only in the maximal test but not in the minimal pain threshold test. Because the minimal pain threshold corresponds to the minimal intensity of the physical stimulus that activates the nerve endings that are responsible for the transduction of such stimulus into a neuronal signal, it can be argued that AT does not have an effect at the level of peripheral transduction of the stimulus. On the other hand, because the perception of pain intensity, in particular the perception of the maximal tolerable intensity, involves the modulation of the transmission of pain within the central nervous system, it can be argued that AT has an effect on these mechanisms. From this point of view, our results are in agreement with the principle of reflexology since the stimulation of a precise region on the earlobe might have excited specific brain region increasing pain threshold in the distal correspondent region of the body [2, 9]. However, more functional investigations are required to address this hypothesis, that is, modern functional magnetic resonance imaging techniques. In our experimental condition, we found an increase of the pain threshold only after 24 hours of treatment. Even though we do not provide direct evidence to explain this observation, it is conceivable that higher production and storage of relevant levels of neuropeptides and the release of anti-inflammatory cytokines might be required to increase pain threshold. In fact, it has been demonstrated that the stimulation of acupoints causes the release of neuropeptide-induced anti-inflammatory cytokines in long term effects [27, 28, 43, 44]. Furthermore, in our study, we used* Vaccaria* seeds because auricular point acupressure by botanical plant seeds is a method to deliver AT less frequently employed and not yet validated. Concerning this issue, encouraging results have been obtained by Yeh and collaborators who reported that auricular acupressure was highly accepted by patients affected by chronic low-back pain and reduced pain medication use among them [20]. The above findings were also corroborated by more recent studies indicating that auricular acupressure could reduce pain intensity and analgesic use in cancer patients [45]. In persons diagnosed with dementia, ear acupressure treatment induced beneficial effects on pain, anxiety, and depression [46]. Acupressure treatment also showed a significant improvement in menstrual distress and low-back pain during 12 months of treatment in dysmenorrheic young adult women [47]. Our results indicating that auricular acupressure could modulate pain threshold independently from the presence of disease confirm the possibility to adopt this method in pain management. Indeed, although auricular acupressure is administered at acupoints that are the same of those of acupuncture, it is generally expected that acupressure is less efficacious than acupuncture on pain relief. This is probably due only to the more strength of evidence available for acupuncture compared to auricular acupressure [20]. In terms of the efficacy of the different treatment methods, some authors, analyzing randomized controlled trials, have recently showed that auricular acupressure exhibits the largest strength of evidence for pain relief, followed by auricular acupuncture [20]. Anyway, both auricular acupuncture and acupressure have the undeniable advantages of low cost and noninvasiveness (also compared with body acupuncture) and particularly auricular acupressure is self-managed: once seed is applied by a trained physician, it can remain in place for a long time (up to one month) and patients themselves can stimulate the acupoint by a gentle pressure with the fingers.

*Vaccaria* seeds were applied by us on the top of the scaphoid fossa of one earlobe; although there are differences among different schools of ear map, the finger points are at similar location either in Chinese or in Nogier's somatotopic maps; therefore our data indicating a reduction of pain perception on fingers corroborate the accuracy of the location of the hand and fingers and suggest for the first time that AT by acupressure can modulate pain threshold independently from the type and presence of disease. It is noteworthy that in our experimental condition sham therapy was administered on the neck and not on the ear; this was performed to avoid any increase of the pain threshold in other parts of the body. In fact, according to Nogier's theory [3], AT works on a microsystem basis (not meridian basis such as body acupuncture) with the ear as a self-contained microsystem that can affect corresponding areas of the whole body. Finally, despite the fact that the present investigation has been conducted on a small sample of people, the results of the statistical analysis support the ability of the proposed methodological approach to objectively verify the efficacy of acupressure treatments on pain perception. This preliminary result encourages the proposal and justifies the cost for future large scale investigations about the effects of acupressure and other nonconventional medical treatments (e.g., acupuncture or homeopathic medicaments) on pain using autoalgometry.

## Figures and Tables

**Table 1:** Physical characteristics of subjects' groups.

Subject	Control mean ± S.E.	Treated mean ± S.E.
Age (years)	20.8 ± 0.2	21.6 ± 0.4
Weight (Kg)	66.0 ± 1.7	70.0 ± 3.0
Height (cm)	170.0 ± 2.0	169.0 ± 2.2

**Table 2:** Measures of pain thresholds obtained with the autoalgometer.

Pain threshold	Time (hours)	Control mean ± S.E. (g)	Treated mean ± S.E. (g)
Minimal score	0	378 ± 19	373 ± 42
	1	318 ± 11	276 ± 24
	24	306 ± 12	317 ± 29

Maximal score	0	436 ± 28	372 ± 52
	1	371 ± 24	306 ± 39
	24	331 ± 24	380 ± 51

## Abbreviations

AT: Auriculotherapy
NGF: Nerve growth factor
NF-*κ*B: Nuclear factor-*κ*B
IL: Interleukin
TNF-*α*: Tumor necrosis factor-*α*.
